# Hsa_circ_0026134 expression promoted TRIM25- and IGF2BP3-mediated hepatocellular carcinoma cell proliferation and invasion via sponging miR-127-5p

**DOI:** 10.1042/BSR20191418

**Published:** 2020-07-15

**Authors:** Wei Zhang, Liang Zhu, Guowei Yang, Bo Zhou, Jianhua Wang, Xudong Qu, Zhiping Yan, Sheng Qian, Rong Liu

**Affiliations:** 1 Department of Intervention Radiology, Zhongshan Hospital of Fudan University, No. 180 Fenglin Road, Xuhui, Shanghai 200032, China; 2 Shanghai Institute of Medical Imaging, Shanghai 200032, China

**Keywords:** hepatocellular carcinoma, hsa_circ_0026134, IGF2BP3, miR-127-5p, TRIM25

## Abstract

Increasing evidence shows that circular RNAs (circRNAs) play a regulatory role in cancer. In the present study, we aimed to investigate the characteristics and effects of hsa_circ_0026134 in hepatocellular carcinoma (HCC). We investigated hsa_circ_0026134 expression in 20 pairs of clinical tissues from HCC patients; expression of hsa_circ_0026134 in different cell lines; effect of hsa_circ_0026134 on proliferation and invasion of HCC cell lines; and the regulatory mechanisms and interactions among hsa_circ_0026134, miR-127-5p, tripartite motif-containing protein 25 (TRIM25) and insulin-like growth factor 2 mRNA-binding protein 3 (IGF2BP3). hsa_circ_0026134 expression was increased in HCC samples and cell lines. Down-regulation of hsa_circ_0026134 attenuated HCC cell proliferation and metastatic properties. Micro (mi)RNA (miR)-127-5p was sponged by hsa_circ_0026134. Rescue experiments indicated that inhibition of miR-127-5p expression promoted cell proliferation and invasion even after hsa_circ_0026134 silencing. TRIM25 and IGF2BP3 were targets of miR-127-5p. Overexpression of TRIM25 or IGF2BP3 promoted cell proliferation and invasion in cells overexpressing miR-127-5p. Down-regulation of hsa_circ_0026134 suppressed TRIM25- and IGF2BP3-mediated HCC cell proliferation and invasion via promotion of miR-127-5p expression, which have been confirmed by luciferase reporter assay. The present study provides a new treatment target for HCC.

## Introduction

Hepatocellular carcinoma (HCC) is the most common primary hepatic malignancy and is a serious health problem worldwide [1,2]. It is the fifth most common cancer and the third leading cause of cancer-related deaths globally [3]. The molecular mechanisms involved in the initiation and progression of HCC are complex and multistep. Further knowledge of these mechanisms would be valuable in predicting prognosis and for the design of more effective therapeutic approaches. Increasing evidence shows that noncoding RNA (ncRNA) plays an important role in regulation of tumorigenesis. ncRNA including miRNA, long ncRNA (lncRNA) and circular RNA (circRNA) [4–6]. miRNA and lncRNA have been extensively studied in various tumors, such as glioblastoma [7], gastric cancer [8], liver cancer [9], colorectal cancer [10] and bladder cancer [11]. However, their role in oncogenesis and development of circRNA is still largely unclear.

circRNAs have been confirmed to be suitable molecular biomarkers for human cancer. Because of their closed structure, circRNAs have high stability and strong resistance to RNA-degradative pathways [12]. Many reports have identified differentially expressed circRNAs in HCC, for example, expression of hsa_circ_103809 suppresses HCC proliferation and invasion by sponging miR-620 [13]. hsa_circ_0078602 can be used as a prognostic biomarker for patients with HCC [14]. hsa_circ_0079929 expression inhibits tumor growth in HCC [15]. Previous studies have found that hsa_circ_0026134 regulates non-small cell lung cancer cell proliferation and invasion via sponging miRNA (miR)-1256 and miR-1287 [16]. However, the role of hsa_circ_0026134 in HCC is still unclear.

The aim of the present study was to elucidate the role and regulatory mechanism of hsa_circ_0026134 in proliferation and invasion of HCC. Hopefully, hsa_circ_0026134 may be useful as a prognostic biomarker and therapeutic target against HCC.

## Materials and methods

### Cells lines and cell culture

Liver cancer cell lines (HepG2, HepaRG, LM3 and SK-Hep1) were provided by the cell bank of the Chinese Academy of Sciences and the normal human hepatic cell line (LO2) was preserved in our laboratory and maintained in RPMI-1640 supplemented with 10% fetal bovine serum (FBS; Gibco, U.S.A.), 100 U/ml penicillin, and 100 μg/ml streptomycin at 37°C in a 5% CO_2_ incubator.

### Cell transfection

The miR-127-5p mimics and the negative control, siRNA against hsa_circ_0026134, and miR-127-5p inhibitor were synthesized by GenePharma (Shanghai, China) and transfected into the HepG2 and HepaRG cells to a final oligonucleotide concentration of 20 nmol/l. The full-length tripartite motif-containing protein 25 (TRIM 25) and insulin-like growth factor 2 mRNA-binding protein 3 (IGF2BP3) were obtained from a human cDNA library and ligated into pCDNA3.1 vector. All cell transfections were introduced by Lipofectamine® 3000 (Invitrogen Life Technologies, U.S.A.). For each cell transfection, three replicates were performed.

### Transwell assay

The cells were transfected with siRNA against hsa_circ_0026134, miR-127-5p mimic, miR-127-5p inhibitor, TRIM25 overexpression vector, and IGF2BP3 overexpression vector. After 48 h, the cells were starved in medium without serum for another 12 h, digested with trypsin, and seeded in the top chambers of 24-well Transwell culture inserts (Promega, Madison, WI, U.S.A.). The medium supplemented with 20% serum was used as a chemoattractant. After 24 h, the cells were fixed for 10 min with 4% formalin.

### Quantitative reverse transcription-polymerase chain reaction

Total RNA was extracted from tissues and cells using TRIzol reagent (Invitrogen, Carlsbad, CA, U.S.A.). cDNA was synthesized and amplified using the TaqMan miRNA Reverse Transcription Kit. The mRNA levels of hsa_circ_0026134, TRIM25, IGF2BP3, miR-127-5p, GAPDH and U6 were determined by quantitative reverse transcription-polymerase chain reaction (qRT-PCR) using TaqMan Human miRNA Assay Kit. The 2^−ΔΔ*C*_T_^ method was utilized to measure the relative fold difference. The hsa_circ_0026134, TRIM25, IGF2BP3, miR-127-5p, GAPDH and U6 primers for PCR were designed by GenePharma (Shanghai, China).

### Cell proliferation and clone formation assays

The Cell Counting Kit-8 (CCK-8) assay was used to detect cell proliferation. Transfected cells were seeded into 96-well plates at 2000 cells/well in triplicate wells. Cell viability was measured by the CCK-8 system (Invitrogen) at 0, 24, 48 and 72 h after seeding. The values were examined by a microplate reader at 450 nm. For the colony formation assay, transfected cells were seeded into six-well plates at 2000 cells/well and maintained in RPMI-1640 medium containing 10% FBS for 10 days. The colonies were imaged and counted after they were fixed and stained.

### Dual-luciferase reporter assay

We constructed reporter plasmids containing wildtype Luc-hsa_circ_0026134/TRIM25/IGF2BP3 (WT) and mutant Luc-hsa_circ_0026134/TRIM25/IGF2BP3. miR-127-5p mimics were synthesized by GenePharma. We tested the luciferase activity of the indicated cells by Dual-Luciferase Reporter Assay System (Promega) 24 h after transfection.

### HepG2 xenograft model

Ethics Committee in Zhongshan Hospital of Fudan University School of Medicine approved all experiments performed in animals. Male BALB/c nude mice, aged 6–8 weeks, were obtained from the Chinese Academy of Sciences (Beijing, China) and housed under standard conditions in laboratory of Zhongshan Hospital of Fudan University School of Medicine in accordance with approved protocols. Following acclimation, the right flank of each experimental mouse was subcutaneously injected with HepG2 cells (2 × 10^6^) suspended in PBS (200 μl). Tumor volumes were measured every 5 days. After 4 weeks, the mice were killed (mice were anesthetized via an intraperitoneal injection of sodium pentobarbital (30 mg/kg) before killing by a dislocated neck). Tumor volume (V) was calculated as V = (length * width^2^)/2.

### Statistical analysis

Continuous variables were expressed as the mean ± standard deviation (SD). One-way analysis of variance was performed for multiple comparisons using GraphPad Prism version 5.0 (GraphPad, La Jolla, CA, U.S.A.). *P*≤0.05 indicated a statistically significant difference.

## Results

### Enhanced expression of hsa_circ_0026134 in HCC tissue and cell lines

**Figure 1:**
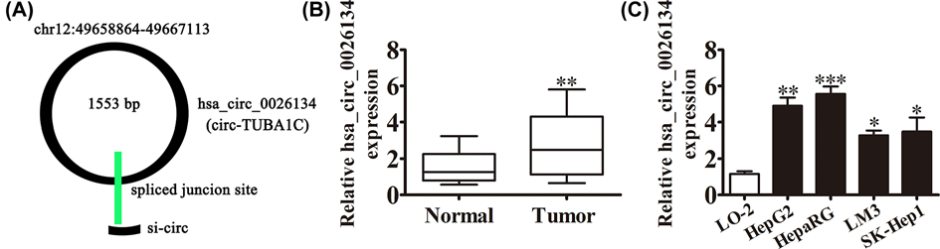
Chromosomal localization and relative expression of hsa_circ_0026134 in HCC tissue and cell lines (**A**) Schematic diagram of the genomic location and structure of hsa_circ_0026134. (**B**) Relative expression of hsa_circ_0026134 in 20 pairs of HCC tissue samples and adjacent noncancerous tissue samples measured by qRT-PCR. Data are expressed as mean ± SD. **P<0.001 versus Normal. (**C**) Relative expression of hsa_circ_0026134 in HCC cell lines and normal cell line measured by qRT-PCR. Data are expressed as mean ± SD. *P<0.05, **P<0.01, ***P<0.001.

Bioinformatics analysis (http://www.circbase.org/cgi-bin/simplesearch.cgi) found that hsa_circ_0026134 was located at chr12:49658864-49667113. hsa_circ_0026134 was cyclized by part of an exon from *TUBA1C* gene. So, hsa_circ_0026134 was also named circRNA TUBA1C (Figure 1A). qRT-PCR detection showed that expression of hsa_circ_0026134 in HCC samples was significantly increased when compared with their normal counterparts (Figure 1B). qRT-PCR uncovered that hsa_circ_0026134 level was markedly enhanced in HepG2, HepaRG, LM3 and SK-Hep1 cells relative to the human normal epithelium cell line (LO-2) (Figure 1C). It is suggested that hsa_circ_0026134 plays an oncogenic role in HCC cells.

### Down-regulation of hsa_circ_0026134 expression inhibits proliferation and migration of HCC cells

**Figure 2:**
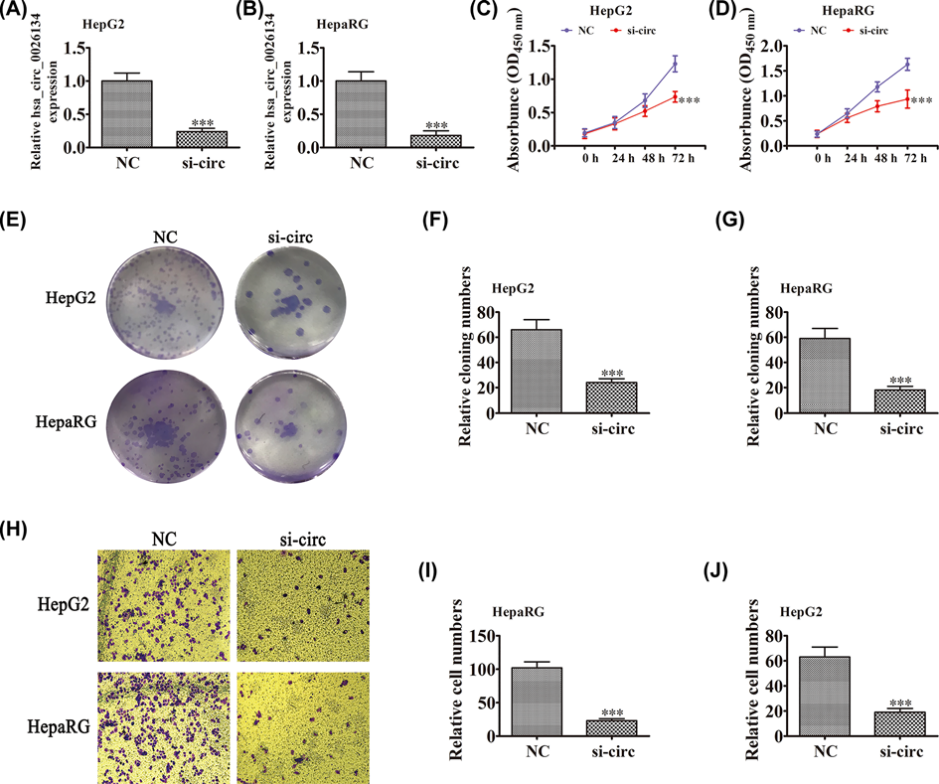
hsa_circ_0026134 silencing inhibits the proliferation and migration of HCC cells *in vitro* (**A,B**) Expression of hsa_circ_0026134 in HepG2 and HepaRG cells was down-regulated by transfection with siRNA against hsa_circ_0026134. Data are expressed as mean ± SD. ***P<0.001 versus negative control (NC). (**C**–**G**) CCK-8 (C,D) and colony formation (E–G) assays showed that hsa_circ_0026134 silencing inhibited growth of HepG2 and HepaRG cells. Data are presented as mean ± SD. ***P<0.001 versus NC. Data are presented as mean ± SD. ***P<0.001 versus NC. (**H–J**) Transwell detection showed that knockdown of hsa_circ_0026134 inhibited migration of HepG2 and HepaRG cells. Data are presented as mean ± SD. ***P<0.001 versus NC. Abbreviation: NC, normal control.

In order to uncover the role of hsa_circ_0026134 in HCC cells, we constructed siRNA against hsa_circ_0026134. qRT-PCR showed that down-regulation of hsa_circ_0026134 significantly suppressed hsa_circ_0026134 expression in HepG2 and HepaRG cells (Figure 2A,B). CCK-8 detection showed that hsa_circ_0026134 silencing suppressed proliferation of HepG2 and HepaRG cells (Figure 2C,D). Colony formation assays showed that hsa_circ_0026134 silencing suppressed proliferation of HepG2 and HepaRG cells (Figure 2E–G). Transwell experiments showed that hsa_circ_0026134 silencing decreased migratory capacity of HepG2 and HepaRG cells (Figure 2H–J). This suggests that down-regulation of hsa_circ_0026134 decreases cell proliferation and migration potential.

### hsa_circ_0026134 can sponge miR-127-5p and regulate TRIM25 and IGF2BP3 expression

**Figure 3:**
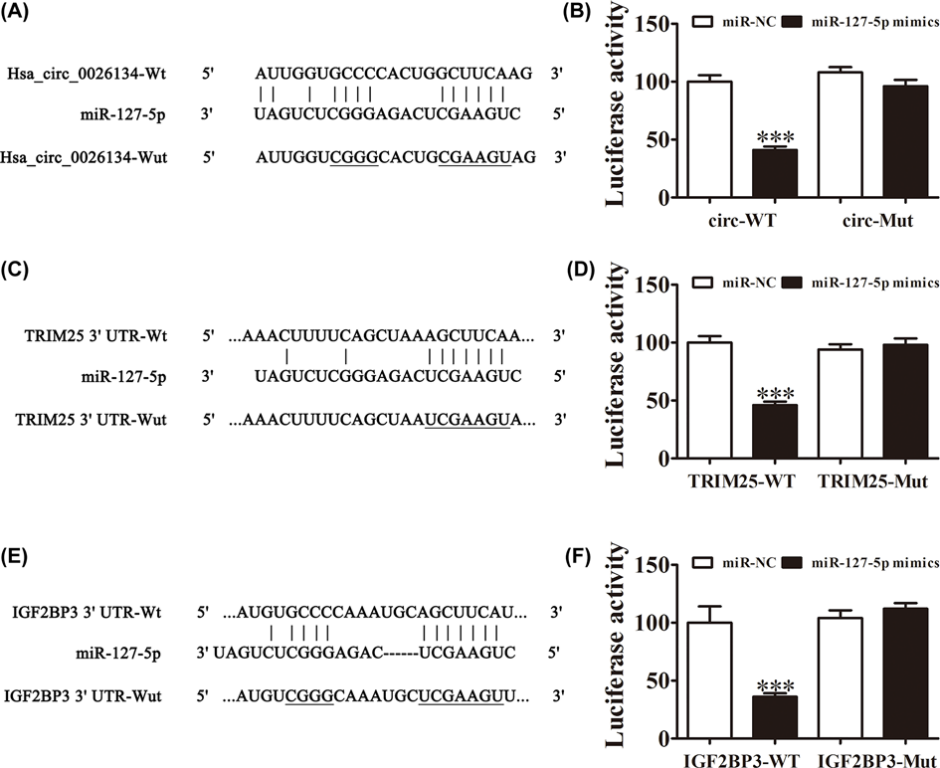
hsa_circ_0026134 sponges miR-127-5p and regulates TRIM25 and IGF2BP3 expression (**A**) Diagrammatic sketch of the binding sites for hsa_circ_0026134 and miR-127-5p. (**B**) Luciferase reporter assay was conducted to evaluate the interaction ability between hsa_circ_0026134 and miR-127-5p. ***P<0.001. (**C**) Correlation analysis of miR-127-5p and the 3′ UTR of TRIM25. (**D**) Luciferase reporter assay was conducted to evaluate the interaction between miR-127-5p and the 3′ UTR of TRIM25. ***P<0.001. (**E**) Correlation analysis of miR-127-5p and the 3′ UTR of IGF2BP3. (**F**) Luciferase reporter assay was conducted to evaluate the interaction between miR-127-5p and the 3′ UTR of IGF2BP3. ***P<0.001.

The miRNAs (miR-1178, miR-1243, miR-1299, miR-142-3p, miR-149, miR-155, miR-197 and miR-127-5p) that may be sponged by hsa_circ_0026134 were predicted by Circular RNA Interactome (https://circinteractome.nia.nih.gov/bin/mirnasearch). Bioinformatics analysis found that only miR-127-5p had a conservative combination with hsa_circ_0026134 (Figure 3A). The luciferase reporter assay indicated that hsa_circ_0026134 inhibited luciferase activity in wildtype but not in mutated cell lines (Figure 3B). Bioinformatics analysis also indicated that TRIM25 and IGF2BP3 were miR-127-5p targets, and miR-127-5p directly interacted with both the 3′ untranslated regions (UTRs) of TRIM25 and IGF2BP3 to suppress mRNA expression (Figure 3C,E). miR-127-5p inhibited luciferase activity in wildtype but not mutant cell lines (Figure 3D,F). The combined results indicated that hsa_circ_0026134 silencing inhibited HCC cell proliferation and migration by targeting the miR-127-5p/TRIM25 and miR-127-5p/IGF2BP3 axis.

### Down-regulation of hsa_circ_0026134 suppresses cell proliferation and migration by recovery of function of miR-127-5p

**Figure 4:**
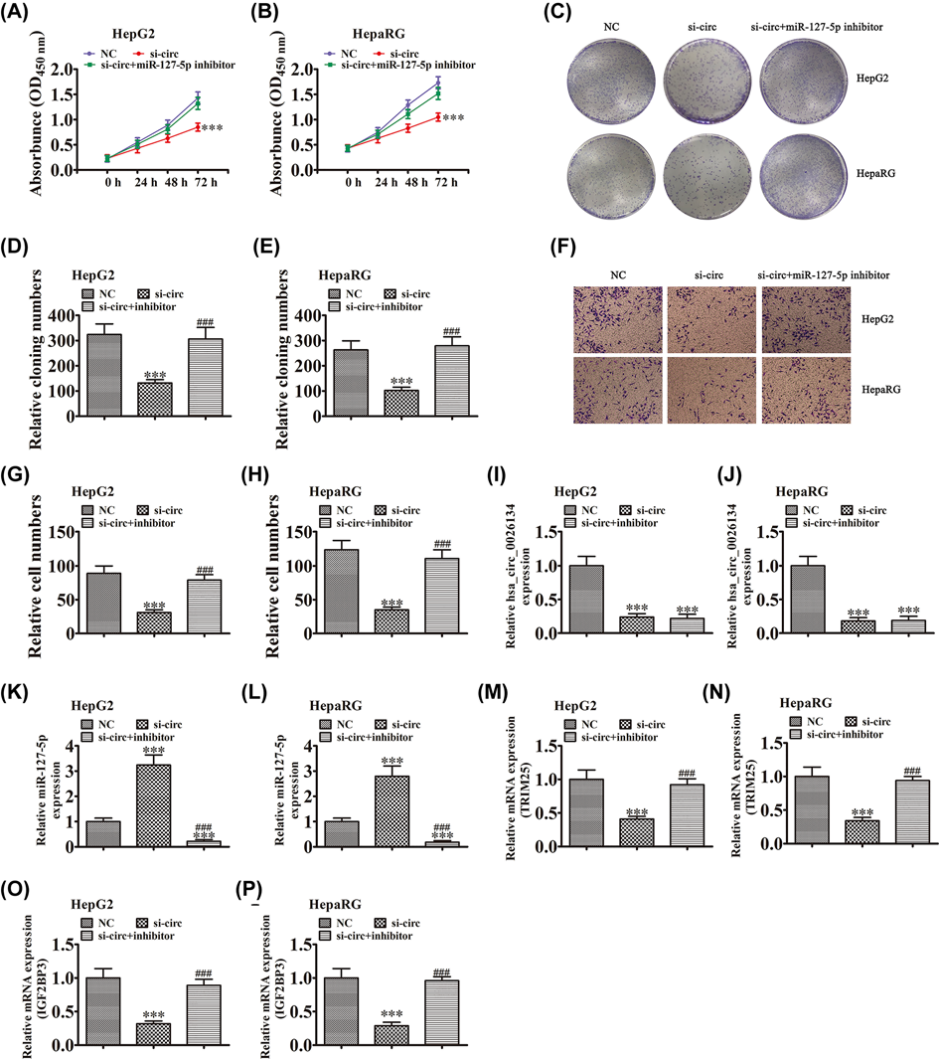
Down-regulation of hsa_circ_0026134 suppressed cell proliferation and migration by recovery of the function of miR-127-5p (**A–E**) CCK-8 (A,B) and colony formation (C–E) assays showed that interaction between hsa_circ_0026134 and miR-127-5p played an important role in regulation of HepG2 and HepaRG cell growth. Data are presented as mean ± SD. ***P<0.001 versus NC. ^###^*P*<0.001 versus siRNA against hsa_circ_0026134 (si-circ). (**F–H**) Transwell detection showed that interaction between hsa_circ_0026134 and miR-127-5p played an important role in migration of HepG2 and HepaRG cells. Data are presented as mean ± SD. ***P<0.001 versus NC. ^###^*P*<0.001 versus si-circ. (**I**–**P**) qRT-PCR detection showed expression of hsa_circ_0026134 (I,J), miR-127-5p (K,L), TRIM25 (M,N) and IGF2BP3 (O,P). Data are presented as mean ± SD. ***P<0.001 versus NC. ^###^*P*<0.001 versus si-circ.

To uncover the regulatory relationship between hsa_circ_0026134 and miR-127-5p, HepG2 and HepaRG cells were transfected with siRNA against hsa_circ_0026134 and miR-127-5p inhibitor. CCK-8 (Figure 4A,B) and colony formation (Figure 4C–E) assays showed that down-regulation of miR-127-5p recovered the proliferative ability of HepG2 and HepaRG cells after down-regulation of hsa_circ_0026134. Transwell detection also showed that miR-127-5p inhibitor treatment recovered the migratory ability of HepG2 and HepaRG cells after down-regulation of hsa_circ_0026134 (Figure 4F–H). qRT-PCR detection showed that hsa_circ_0026134 expression was significantly decreased after transfection with siRNA against hsa_circ_0026134 in HepG2 and HepaRG cells, and miR-127-5p inhibitor treatment had no effect on hsa_circ_0026134 expression after hsa_circ_0026134 silencing (Figure 4I,J). qRT-PCR detection of miR-127-5p showed that down-regulation of hsa_circ_0026134 promoted miR-127-5p expression, but miR-127-5p inhibitor treatment significantly suppressed miR-127-5p expression (Figure 4K,L). qRT-PCR detection of TRIM25 and IGF2BP3 expression found that down-regulation of hsa_circ_0026134 suppressed TRIM25 and IGF2BP3 expression in HepG2 and HepaRG cells, but miR-127-5p inhibitor treatment recovered expression of TRIM25 and IGF2BP3 (Figure 4M–P). It is suggested that TRIM25 and IGF2BP3 expression can be directly regulated by miR-127-5p, and miR-127-5p can be regulated by hsa_circ_0026134.

### Overexpression of TRIM25 or IGF2BP3 promotes cell proliferation and migration

**Figure 5:**
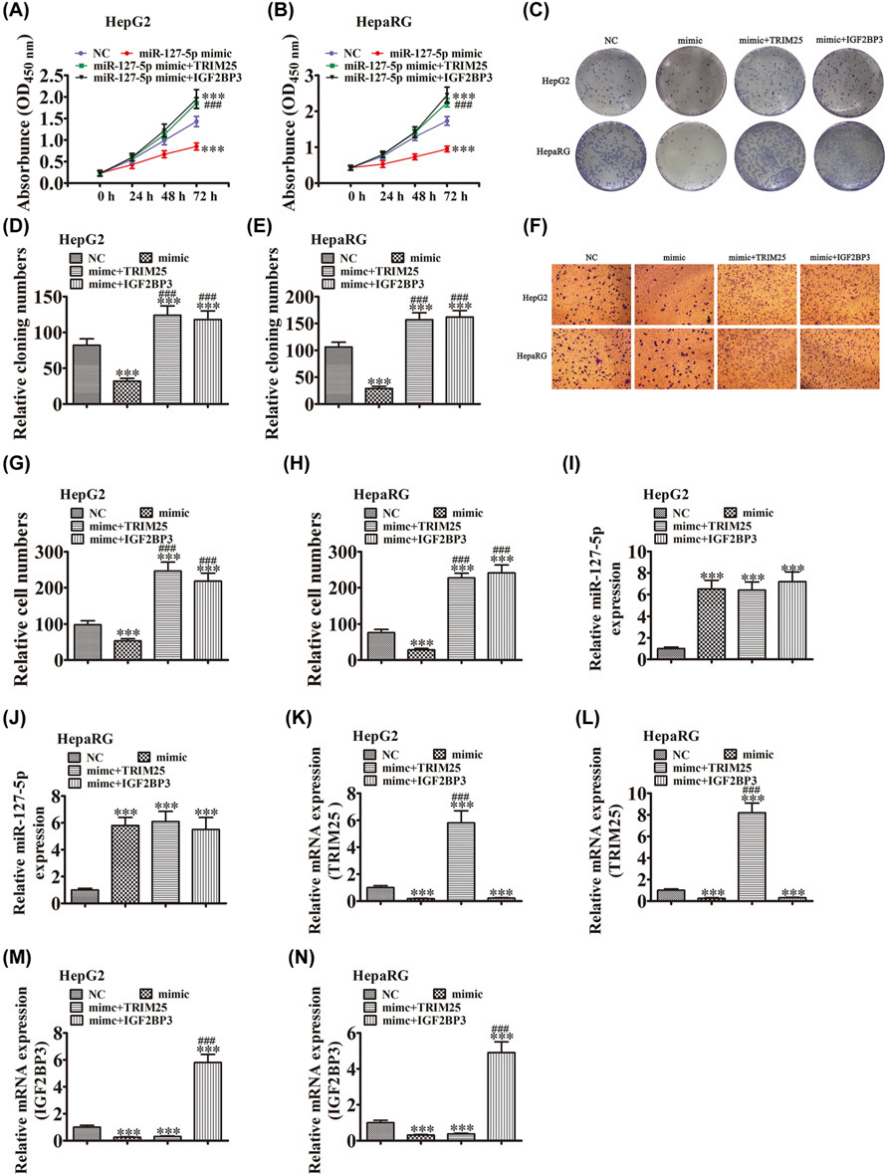
Overexpression of TRIM25 or IGF2BP3 promoted cell proliferation and migration (**A–E**) CCK-8 (A,B) and colony formation (C–E) assays showed that interaction among miR-127-5p, TRIM25 and IGF2BP3 played an important role in growth of HepG2 and HepaRG cells. Data are presented as mean ± SD. ***P<0.001 versus NC. ^###^*P*<0.001 versus miR-127-5p mimic. (**F**–**H**) Transwell detection showed that interaction among miR-127-5p, TRIM25 and IGF2BP3 played an important role in migration of HepG2 and HepaRG cells. Data are presented as mean ± SD. ***P<0.001 versus NC. ^###^*P*<0.001 versus miR-127-5p mimic. (**I**–**N**) qRT-PCR detection showed expression of miR-127-5p, TRIM25 and IGF2BP3. Data are presented as mean ± SD. ***P<0.001 versus NC. ^###^*P*<0.001 versus miR-127-5p mimic.

To elucidate the regulatory relationship among miR-127-5p, TRIM25 and IGF2BP3, HepG2 and HepaRG cells were transfected with miR-127-5p mimic and TRIM25 or IGF2BP3 overexpression vector. CCK-8 (Figure 5A,B) and colony formation (Figure 5C–E) assays showed that overexpression of miR-127-5p decreased the proliferative ability of HepG2 and HepaRG cells. TRIM25 or IGF2BP3 overexpression recovered the proliferative ability of HepG2 and HepaRG cells after up-regulation of miR-127-5p. Transwell detection also showed that TRIM25 or IGF2BP3 overexpression recovered the migratory ability of HepG2 and HepaRG cells after overexpression of miR-127-5p (Figure 5F–H). qRT-PCR detection showed that miR-127-5p expression was significantly increased after transfection with miR-127-5p mimic in HepG2 and HepaRG cells, and TRIM25 or IGF2BP3 overexpression had no effect on miR-127-5p expression (Figure 5I,J). qRT-PCR detection of TRIM25 and IGF2BP3 showed that up-regulation of miR-127-5p decreased TRIM25 and IGF2BP3 expression. Expression of TRIM25 and IGF2BP3 was significantly increased in HepG2 and HepaRG cells after transfection with TRIM25 or IGF2BP3 overexpression vector (Figure 5K–N). These results suggest that TRIM25 and IGF2BP3 expression can be directly regulated by miR-127-5p.

### Down-regulation of hsa_circ_0026134 suppressed the HepG2 tumor formation

**Figure 6:**
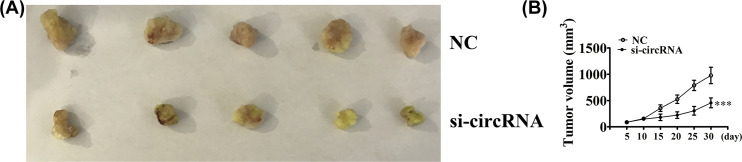
Down-regulation of hsa_circ_0026134 inhibited the growth of HCC cells *in vivo* (**A**) Representative photographs of HepG2 tumor formation in the xenografts of nude mice. (**B**) Summary of the tumor volume in mice measured weekly. Data are presented as the mean ± SD. ***P<0.001 vs. NC.

The mouse xenograft model of HepG2 tumor formation demonstrated that down-regulation of hsa_circ_0026134 suppressed tumor growth, as compared withuntreated control cells (Figure 6A,B).

## Discussion

Since the demonstration of the stable expression of numerous circRNAs in eukaryotic cells and the fact that some of them possess strong miRNA-binding capability, interest in the role of circRNAs in a variety of diseases has risen [17,18]. circRNA profiling has identified circADAMTS13 as an miR-484 sponge that suppresses cell proliferation in HCC [19]. In this study, hsa_circ_0026134 was highly expressed in HCC compared with adjacent tissue, and in HCC cell lines. hsa_circ_0026134 knockdown inhibited proliferation and migration of HCC cells. A previous study using microarray profile analysis found that hsa_circ_0026134 was overexpressed in non-small cell lung cancer. hsa_circ_0026134 expression promotes cell progression through sponging miR-1256 and miR-1287 [16]. In this study, we found that expression of hsa_circ_0026134 was also increased in HCC. Down-regulation of hsa_circ_0026134 suppressed proliferation and migration of HCC cells by recovery of the function of miR-127-5p. Bioinformatics and luciferase reporter assays found that miR-127-5p was the target of hsa_circ_0026134. It is reported that miR-127-5p suppresses growth of HCC cells by targeting the biliverdin reductase B/nuclear factor-κB pathway [20]. miR-127 suppresses gastric cancer cell migration and invasion via targeting Wnt7a [21]. It is suggested that miR-127 has anticancer effects. Down-regulation of miR-127-5p recovered the proliferation and migration in HCC cells after hsa_circ_0026134 silencing. Previous studies have found that miR-127-5p can be regulated by hsa_circ_0001649. The study found that was markedly decreased in HCC lines and tumor tissues. Overexpression of hsa_circ_0001649 greatly inhibited proliferation and migration of HCC by sponge miR-127-5p, miR-612 and miR-4688 [22]. Suggestion that miR-127-5p an important role in medaiting the progression of HCC.

Our further study found that miR-127-5p interacted with the 3′ UTRs of TRIM25 and IGF2BP3. Overexpression of TRIM25 or IGF2BP3 can reverse the anticancer effect of miR-127-5p. TRIM25, also known as estrogen-responsive finger protein, is up-regulated in HCC [23], prostate cancer [24], and non-small-cell lung carcinoma [25]. Down-regulation of TRIM25 has an anticancer effect. IGF2BP3, also known as IMP3, belongs to a conserved IGF2 mRNA-binding protein family. IGF2BP3 was first identified due to its high abundance in pancreatic carcinoma [26]. After its initial identification, IGF2BP3 was found to be a mainly overexpressed member of its protein family in various tumor types, such as squamous cell carcinoma [27], lung cancer [28], melanoma [29], colon cancer [30] and liver cancer [31]. It has also been found that IGF2BP3 can stabilize TRIM25, and both TRIM25 and IGF2BP3 play an essential role in cancer cell proliferation [32,33]. We found that hsa_circ_0026134 expression promoted TRIM25- and IGF2BP3-mediated HCC cell proliferation and invasion via sponging miR-127-5p.

In conclusion, we observed that hsa_circ_0026134 up-regulation in HCC was associated with tumor progression. Our results demonstrated that hsa_circ_0026134 influenced the malignant behavior of HCC cells via the miR-127-5p/IGF2BP3 and miR-127-5p/TRIM25 regulatory networks, suggesting that hsa_circ_0026134 is a potential therapeutic target.

## Abbreviations

CCK-8: cell counting kit-8
circRNA: circular RNA
FBS: fetal bovine serum
HCC: hepatocellular carcinoma
IGF2BP3: insulin-like growth factor 2 mRNA-binding protein 3
lncRNA: long noncoding RNA
ncRNA: noncoding RNA
qRT-PCR: quantitative reverse transcription-polymerase chain reaction
TRIM25: tripartite motif-containing protein 25
UTR: untranslated region
