# Expression of Concern: Hsa_circ_0026134 expression promoted TRIM25- and IGF2BP3-mediated hepatocellular carcinoma cell proliferation and invasion via sponging miR-127-5p

**DOI:** 10.1042/BSR20191418_EOC

**Published:** 2026-05-06

**Authors:** Wei Zhang, Liang Zhu, Guowei Yang, Bo Zhou, Jianhua Wang, Xudong Qu, Zhiping Yan, Sheng Qian, Rong Liu

**Affiliations:** 1 Department of Intervention Radiology, Zhongshan Hospital of Fudan University, No. 180 Fenglin Road, Xuhui, Shanghai 200032, China; 2 Shanghai Institute of Medical Imaging, Shanghai 200032, China

**Keywords:** hepatocellular carcinoma, hsa_circ_0026134, IGF2BP3, miR-127-5p, TRIM25

*Bioscience Reports* has been made aware of potential issues surrounding the scientific validity of this paper and is hence issuing a permanent Expression of Concern to notify readers.

A duplicated region has been identified between two panels of Figure 5F within this article, and it has been noted that there is a difference in colour between the two regions of similarity.

When first contacted regarding the concerns, the authors provided raw data, which contained no additional concerns. Replacement data was also provided. However, no explanation has been provided for the changes in colour between the two regions, and the authors have not responded to further contact.

Given the unresolved nature of this concern, the Editorial Board has decided to issue a permanent Expression of Concern for this article.

